# Self-protecting motivation, indexed by self-threat, modifies retrieval-induced-forgetting and confidence in employment decision bias against out-group targets

**DOI:** 10.1186/s41235-021-00334-w

**Published:** 2021-12-11

**Authors:** Shaohang Liu, Christopher Kent, Josie Briscoe

**Affiliations:** School of Psychological Science, University of Bristol, 12a Priory Road, Clifton, Bristol, BS8 1TU UK

**Keywords:** Ethnicity, Retrieval-induced forgetting, Self-threat, Motivated cognition, Stereotypes

## Abstract

Human memory is malleable by both social and motivational factors and holds information relevant to workplace decisions. Retrieval-induced forgetting (RIF) describes a phenomenon where retrieval practice impairs subsequent memory for related (unpracticed) information. We report two RIF experiments. Chinese participants received a mild self-threat manipulation (Experiment 2) or not (Experiment 1) before an ethnicity-RIF task that involved practicing negative traits of either in-group (Chinese) or an out-group (Japanese) target. After a subsequent memory test, participants selected their preferred applicant for employment. RIF scores correspond to forgetting of unpracticed positive traits of one target (Rp−) relative to the recall of practiced negative traits of the other target (Rp+). Enhanced forgetting of positive traits was found in both experiments for both targets. Across experiments, a significant target by threat interaction showed that target ethnicity modified RIF (an ethnicity-RIF effect). Inducing a self-protecting motivation enhanced RIF effects for the out-group (Japanese) target. In a subsequent employment decision, there was a strong bias to select the in-group target, with the confidence in these decisions being associated with RIF scores. This study suggests that rehearsing negative traits of minority applicants can affect metacognitive aspects of employment decisions, possibly by shaping the schemas available to the majority (in-group) employer. To disrupt systemic racism, recruitment practices should aim to offset a human motivation to protect one-self, when exposed to a relatively mild threat to self-esteem. Discussing the negative traits of minority applicants is a critical, and sensitive, aspect of decision-making that warrants careful practice. These data suggest that recruiting individuals should be reminded of their personal strengths in this context, not their vulnerabilities, to secure their decision-making for fairer recruitment practice.

## Significance statement

Systemic racism refers to how structures of power and influence in society (e.g., education, judicial, political, or workplace systems) retain and promote disadvantage and inequality for some people due to their color, culture, or ethnic origin. Identifying cognitive pathways to biased decision-making within the workplace is essential to help protect people from disadvantage and inequality. This is most relevant during recruitment and promotion activity where positive and negative attributes of ethnic minority candidates need to be openly evaluated and discussed. In these settings, candidate identity becomes relevant to memory and decision-making. Retrieval-induced forgetting (RIF) is a memory phenomenon that is modified by social stereotypes in Western societies. The present study used a self-threat induction task, followed by a RIF paradigm, and an employment decision task, to find out how Chinese people’s motivations can influence forgetting and decision-making. Practicing negative traits of a target person (either Chinese or Japanese) influenced the subsequent forgetting of their positive (unpracticed) traits; a RIF effect. Across two experiments, RIF scores were found to be modified by the ethnic identity of the out-group (Japanese) target. This ethnicity-RIF effect was only observed when Chinese participants experienced a mild threat to their self-esteem by comparing unfavourably to other people in a previous induction task. Being motivated to enhance and protect oneself can change what is forgotten about other people. Chinese people were strongly biased to select the Chinese applicant for employment, but their confidence in this decision related to their forgetting of the Japanese applicant’s traits under conditions of self-threat. These findings highlight the risks to minority out-group applicants when their personal traits are rehearsed and retrieved in a decision-making context. Ethnicity becomes more relevant to memory and decision-making when people are motivated to protect their own self-esteem. To disrupt systemic racism, we recommend that recruitment practice should include reminders to people in decision-making roles of their personal strengths, rather than emphasizing their vulnerabilities, especially in how they compare favourably to other people. Such reminders should be positioned carefully in relation to evaluation of, and discourse about, negative traits of the applicant. This practice would off-set self-protecting motivations that enhance selective forgetting of personal traits of ethnic minority candidates and shapes the schemas used in social exchange and decision-making. Overall, we highlight how cognitive inhibition is malleable to personal motivation, giving opportunity for developing cognitive tools that will combat systemic racism within the workplace.

## Introduction

To understand and address systemic racism, one needs to acknowledge the inadvertent nature of bias within the dialogue, policies and processes of institutional decision-making. Cognitive bias is often associated with conceptual stored knowledge, derived from misplaced inferences or illusory correlations, and is typically described in relation to schema (stereotype) activation. This knowledge has been linked to the cumulative and probabilistic frequency of associations that build from a wider cultural experience of different racial and ethnic groups (so-called ‘culture in mind’, Hinton, 2017). Even if culture ‘in mind’ reflects the maintenance of cultural concepts relating to ethnicity, controlled cognition also contributes to the regulation of concept knowledge (Anderson & Hulbert, 2021; Badre & Wagner, 2007). To address systemic racism is to examine the cognitive mechanisms that actively lead to biased decision-making, not just to highlight the formation of cultural concepts about social groups. The current study builds on the idea that memory, specifically the mechanisms of forgetting (as a memory ‘modifier’; Bjork, 1975) is one cognitive pathway to inadvertent bias in decision-making. Biased decisions in the workplace are particularly harmful, by restricting opportunity for recruitment or promotion. Such decisions occur in a time-limited context (for example, post-interview) that requires active rehearsal of, and social exchange about, the personal traits of different people, often alongside contextual cues to their individual and ethnic identity. It is important to determine when, and how, remembering and forgetting information about other people, will influence the evaluation of self and others in the workplace, as a pathway to systemic racism.

Culturally-bound information about ethnic groups occurs over a developmental time-frame of long-term exposure (Hinton, 2017). Over relatively short time-frames, the availability of person-specific knowledge can generate schemas, as more agile patterns of knowledge activation. Forgetting is especially relevant as it allows for the *selective* retrieval of information about other people. Forgetting changes the nature of schemas held about other people, by altering the amount and quality of information that becomes available. Schemas act as a cognitive filter to the social exchange of information about cultural groups and can be instrumental to the decisions made. Retrieval-induced forgetting (RIF) is a specific phenomenon that refers to enhanced forgetting for material related to memory targets, after those targets have previously been retrieved. A standard paradigm was first proposed by Anderson et al. (1994) consisting of three phases. In the initial study phase, participants learned category-exemplar pairs taken from different categories. Then, in the retrieval practice phase, about half of the exemplars of some (not all) of the category lists were recalled. This manipulation generates three within-subject conditions: retrieved exemplars in the retrieved category (as Rp+), unretrieved exemplars from those same retrieved categories (as Rp−) and unretrieved exemplars in the unretrieved categories (Baseline-BL). Finally, all the exemplars are tested. The typical pattern of memory retrieval using this paradigm is a mnemonic advantage for recalling practiced (Rp+) exemplars and a relative disadvantage for recalling related (Rp−) exemplars, as compared to recalling the BL items.

The dominant explanation of forgetting in the RIF paradigm is an inhibitory control account (Anderson, 2003). The act of recalling Rp+ items triggers an automatic process of inhibition of the RP− items that stems from the interference of a related category cue. BL exemplars are cued from a different category, and therefore do not correspond to the categories being suppressed. Ostensibly, eliciting RIF effects appears to depend on a cue to group identity, whether the category distinguishes the exemplars of vegetables (from fruits), women (from men) or a Spanish (from German) person. In standard verbal paradigms, when participants are provided with a category cue (e.g. Spanish), then related, but unpracticed, exemplars of a target category (e.g. Santiago; a Spanish name) are inhibited/suppressed relative to exemplars from an unpracticed baseline category (e.g. Klaus; a German name). Closer examination of RIF effects reveals that competition between Rp+ and Rp− items is critical to the selection of the successfully retrieved items at test (see Anderson & Spellman, 1995). For example, using a novel test cue that is independent of the category (but still semantically related) continues to confer RIF effects at test. This emphasizes how cue identity is not a boundary condition for observing these effects (e.g. Weller et al., 2013). In a social context, it is not the labelling of group identity per se, but the relatedness of traits (e.g. by their valence) that matters. Given co-activation of practiced traits with related (if unpracticed) traits, then these conditions invoke inhibitory control processes to suppress the unpracticed traits. This is effectively a ‘quiet’ form of selective retrieval in how information is brought to mind.

One outstanding issue is whether these RIF effects extend to ethnic categories, as groups with converging perceptual and cultural attributes that signify their identity (e.g., language, gender, musical preference, nationality, residency). Eliciting RIF effects partly depends on the degree of similarity (or family resemblance) between category members (Anderson & McCulloch, 1999). In a series of studies with European (Italian) participants, Pica and colleagues have determined that social categorization by gender, ethnic, and sexual identity can elicit RIF of personal traits (e.g., Pica et al., 2016, 2017, 2018b). A critical pattern observed in social RIF is that the co-activation of culturally-acquired concepts that are associated with group identity can moderate the size of RIF effects; a stereotypic RIF effect. For example, Pica et al., (2018b; Study 2) observed *larger* RIF effects (more forgetting of RP- items relative to BL items) for personal traits that were incongruent with male leadership, when cued by female manager, but relatively *smaller* RIF effects (less forgetting) of traits associated with feminine roles. However, caution is needed to extrapolate any expectation that RIF effects extend in similar ways to the group contrasts between different ethnic categories. The strength of association between the memoranda and the associated stereotype can vary for different social groups. So, using trait memoranda that directly align with a specific stereotype (e.g., male leaders as strong) is different to the illusory association between a stereotype target and negative valence of otherwise indiscriminate personal traits i.e., ‘throwing shade’ at the traits of an individual or group. One ethnicity-RIF study by Pica et al. (2017) addressed forgetting of personal traits linked to an African-American or a European-American job applicant. Participants had to encode personal traits with positive or negative valence as indirectly associated with their identity. They found RIF effects were equable for the positive traits, however, the forgetting (RIF) of negative traits was *smaller* for the African-American applicant, relative to the European-American applicant. It seems these forgetting effects are moderated by other factors related to ethnicity, such as cultural stereotypes, or factors relating to the internal motivations and cognitive resources of the observer.

Motivated cognition offers an alternative account for theorizing about RIF effects (see Pica et al., 2018a for discussion, also Pica et al., 2013, 2014; Pica et al., 2018b). A well-established social motivation is the need for self-enhancement to maintain a positive self-view and encourage higher self-esteem (Sedikides & Gregg, 2008; Sedikides & Green, 2009; Zhang et al., 2018). This can be expressed through *self-advocating* to increase the benefits of being highly regarded (for example, by advocating oneself as superior to others), or by *self-protecting* to diminish the likelihood of receiving a negative view of oneself, for example, by ignoring negative evaluation (Alicke & Sedikides, 2009). At first glance, self-advocating does moderate RIF effects. For example, Macrae and Roseveare (2002) used gift lists as memoranda and found that RIF effects occurred only when the gifts were purchased for others and not when benefitting the participants themselves. Advocating for one’s own personal gain increased the memorization (less forgetting) of purchased gifts. Self-protecting motivations have also been shown to alter the pattern of social RIF effects. In Pica et al.’s (2016) study of homosexual and heterosexual targets, a self-threat manipulation was administered using nonverbal reasoning (IQ) tests followed by moderately positive or negative feedback to participants. For the target from a stigmatized out-group of homosexuals, they found a different pattern of RIF effects after negative feedback. That is, the size of the RIF effect reduced after practicing negative items, and only marginally increased after practicing positive items. No effects of a self-threat manipulation were observed for the in-group target. In this case, the self-threat manipulation instigated a desire to protect self-esteem, by encouraging retrieval of negative traits of the stigmatized target (see also Kunda, 1990; Kunda & Sinclair, 1999).

Motivational influences have been identified as relevant to ethnicity-RIF effects (Dunn & Spellman, 2003; Pica et al., 2017). Pica et al. (2017) interpreted the smaller ethnicity-RIF effect for negative traits of African-American targets as being congruent with negative attitudes held towards African-American people. These effects are similar to the modified social RIF effects induced by a self-protecting motivation with stigmatized (homosexual) targets reported by Pica et al. (2016). In standard accounts of RIF, forgetting arises from inhibitory processes that are triggered by the need for interference control during practice (Levy & Anderson, 2002; Storm & Levy, 2012). When negative traits (as practiced items) are retrieved, there is suppression of competing non-target items (i.e. positive traits) that alters their accessibility in the final test (see also Murayama et al., 2014). However, as noted by Pica et al. (2016), motivated cognition could be compatible with the inhibitory account, if one assumes that inhibition is also goal-directed and orients retrieval to items that are congruent with the observer’s goal state. So, practicing items with a negative valence confers suppression of positive items according to standard inhibitory control accounts, whereas negative traits are enhanced by guided search of memory for stereotype-congruent information, in accordance with a self-protecting motivation.

Understanding the cognitive mechanisms underpinning the ethnicity-RIF have important consequences for combating systemic racism, especially within the workplace. One hallmark of systemic racism is the likelihood of inadvertent bias in decisions about recruitment and promotion with damaging consequences for ethnic minority and other out-group members in terms of their access to, and opportunity within, the workplace. Since recruitment and promotion practices require the personal traits of applicants (both positive and negative traits) to be actively evaluated and discussed, we propose that the ethnicity-RIF gives insight to how valenced information about out-group targets is retrieved. A work-place environment is also likely to encourage self-enhancing and self-protecting motivations amongst employees, who seek to protect their own interests through biased judgement and evaluation of others. Biased decisions involve changing criterion for a given selection, altering the probability of selecting an out-group applicant, or in the metacognitive factors that influence the threshold for a decision, such as decision confidence in appointing or promoting an applicant. If ethnicity-RIF effects alter the accessibility of valenced information related to the rehearsed material, whether, or not, this is due to motivational factors, then this type of forgetting could impact on informational schemas used to evaluate a person from a minority group. The risk is that ethnicity-RIF effects modify schemas by including traits that would otherwise be suppressed, or by omitting traits that would otherwise be retained, biasing the decision-making process.

The primary aim of the current study was to understand ethnicity-RIF effects and their relation to selection bias and decision confidence, as a pathway to inadvertent bias experienced by an out-group minority. As bias occurs at the point of decision-making, this study will determine whether modified forgetting effects in the RIF paradigm, arising from self-protecting motivation, can increase the likelihood and confidence of a dominant (Chinese majority) population selecting an in-group job applicant. To do this, we addressed ethnicity-RIF effects in three ways: first, we considered ethnicity-RIF effects within an East Asian context to ascertain their cross-cultural stability. Second, we focused exclusively on the practice of negative traits in the RIF paradigm, since exchanging valenced information is a critical, and sensitive, aspect of recruitment activity. Third, we manipulated the involvement of metacognitive factors related to self-esteem within the RIF paradigm, to acknowledge the role of motivated cognition.

Across two experiments we addressed two key questions relating to the ethnicity-RIF: First, we asked whether Chinese participants generate ethnicity-RIF effects when remembering negatively-valenced personal traits of Chinese and Japanese job applicants. Following Pica et al. (2017)’s finding of diminished RIF effects for the negative traits of African-American targets, we sought evidence that ethnicity-RIF effects are also generated by majority Han Chinese citizens when evaluating a minority Japanese target. According to inhibitory control accounts, both experiments should elicit competitive activation of (positive) traits of the target applicant that triggers inhibitory control processes, generating RIF effects through the suppression of positive traits. Any modification of the size of these RIF effects linked to target ethnicity, is better explained by motivated cognition. For example, practicing negative traits of a minority out-group target should concur with a search for information that is congruent with existing cultural stereotypes, generating an enhanced RIF from enhanced recall of negative out-group traits, over and above the suppression of positive in-group traits. This provides a possible mechanism for how people inadvertently ‘throw shade’ on the out-group target, in accordance with pre-existing cultural concepts.

Second, we asked whether ethnicity-RIF effects can be linked to a self-protecting motivation (manipulated directly in Experiment 2), especially in the presence of a stigmatized out-group (Chinese) target. Following Pica et al. (2016), we identify self-protecting motivations as linked to their need to restore their self-esteem. However, we also acknowledge that motivations can be elicited or altered in accordance with the ‘in-situ’ experience of threat. Pica et al.’s (2016) study used strong conditions for eliciting self-threat, presented immediately before the retrieval of Rp + items, that was likely to elicit a self-protecting motivation. We used a self-threat induction task to give a ‘softer’ experience of self-threat (i.e. discomfort and ambiguity) by using more neutral feedback (below average performance) on a generic non-IQ task offered at the beginning of the experiment before they reviewed the applicants. For Experiment 1, the feedback provided an above-average score and for Experiment 2, feedback about a below-average score that introduced a threat to their self-esteem. These conditions generalize more closely to the workplace experience where people get mediocre performance feedback with longer spacing before decision-making events. We predicted a modified pattern of ethnicity-RIF-effects in Experiment 2 with more forgetting of positive traits of the Japanese target (as supplemented by guided inhibitory control) when practicing a Chinese target. In accordance with a self-esteem manipulation, we anticipated more recall of positive traits of the in-group target (directly influenced by motivational factors) that could diminish an RIF effect when practicing the Japanese target.

Finally, we sought to explore the relations between scores on the ethnicity-RIF as related to the confidence of the employment decision, since both aspects could be related to meta-cognitive evaluation of self and others.

## Experiment 1

### Methods

#### Participants

One hundred and three Chinese university students were recruited for this experiment (40 females and 63 males, mean age = 21.1 years). Participants received a souvenir medal as a gift for participation. All the participants gave consent to participate in the experiments and we did not adopt any exclusion criterion for the participants. Data was collected by the same experimenter within a short time-frame between December 2019 and January 2020.

#### Design

A mixed design was used where ethnicity of the retrieval practice (Chinese or Japanese applicant) was manipulated between participants, and retrieval type (RP+, RP−, BL) was manipulated within-subject.

To ascertain the cultural stability of ethnicity-RIF effects, we sampled Han Chinese observers of a Chinese—Japanese target. Within the multiple ethnic groups of East Asia, the Japanese and Chinese publics hold longstanding socio-economic and political divisions that arise from a long history of political and economic conflict. Research by the Pew Centre (September, 2016) found explicit and wide-spread attitudes in Chinese and Japanese publics that mutually regarded each other as violent, arrogant, dishonest and not having hardworking traits. In China, these negative attitudes can be widely-shared with only 14% of the Chinese public feeling favourable disposed towards Japan. This gives a likely platform for assimilating cultural knowledge as negative stereotypes of Japanese people by Han Chinese people who comprise a dominant (in-group) in mainland China.

Within the ethnicity-RIF, we focused on practicing negative traits because these are necessary, but sensitive, in the context of social exchanges about recruitment of out-group members. Previous work by Pica et al. (2016) found more robust RIF effects when practicing negative traits, compared to marginal effects with positive traits. So, we aimed for a robust and well-powered study that could identify ethnicity-RIF effects in a different cultural context. Based on a large effect size (*d* > 1.0, as calculated from RP- vs BL difference scores), Pica et al. generated a well-powered ethnicity-RIF effect of *β* = 0.99 with n = 80 American participants. For the current study, the sample size was estimated more cautiously (given a shift in cultural context) as a moderate effect size (0.5) with *β* = 0.80, that required 26 participants for observing reliably replicable effect of RIF. Here, in this study (both experiments), we over-recruited the participants to achieve relatively higher statistical power based on the number of participants that could be recruited within a reasonable timeframe.

#### Material

The learning materials were 24 personal traits (12 positive and 12 negative). In Pica et al. (2016), participants were required to accurately memorize the personal traits of two job applicants, prior to making a recruitment decision to identify the preferred applicant. One limitation of that design was that all traits were considered equally. For example, a lazy person may be a fine friend, but impossible to work with as a colleague in a busy café. The selection of a few traits that confer unemployability, rather than a negative perception of the employee, could retain un-necessary bias in this design. To accommodate this, the current design introduced selected traits that were a priori rated as employable or not employable (even if not strongly desirable in an employee) and were also rated as positive or negatively valenced traits. All participants were further asked to generate perceived confidence of their decision to employ one of the applicants. As bias occurs at the point of decision-making, then this study will determine whether modified forgetting effects in the RIF paradigm, arising from self-enhancing motivation, can increase the likelihood and confidence of a dominant (Chinese majority) population selecting an in-group job applicant.

A pilot study (40 participants) was conducted to obtain ratings of 80 personal traits (40 positive and 40 negatively-valenced traits). Ratings of the negative and positive valence were taken using Likert scales (rated 1 to 5; 1 = very negative; 5 = very positive) and ratings of employability (with Likert scales rated from very 1 to 7; 1 = very unemployable, 7 = very employable). The mean summary of ratings was used to avoid selecting neutral traits (e.g., talkative) and avoid selecting traits that were considered unemployable (e.g., lazy). The selected traits for the two applicants were matched for mean negativity, positivity, and employability (summary data for this pilot study are available here: https://osf.io/49mxw/?view_only=b52482eba300473396293006adb195a5). Additionally, all the traits consisted of two Chinese characters (two syllables) to control for phonological variation in the trait names.

#### Procedure

At the start of the experiment participants took a self-threat induction test based around a semantic association task. Similar to Pica (2016), a self-esteem threat was used by providing negative feedback about the participant’s performance on a neutral semantic test. Over 12 trials, participants were required to generate the most appropriate and related word according to the three words provided as prompts (e.g., prompts: animal, cat, bone; answer: *dog*). Three practice trials were given prior to the formal test. Feedback was provided after each practice trial, but no feedback was given for the formal test trials. For each question, participants had 20 s to type their answer below the prompt words. Critical feedback was provided to the participants immediately after the 12 questions were completed. Participants in Experiment 1 received the feedback “You have correctly answered 9 out of 12 questions. The average score is around 6”. The feedback aimed to highlight metacognitive awareness of their own performance by creating uncertainty regarding their exact score, whilst maintaining a high level of self-esteem regarding their performance in relation to other people.

The Experiment 1 was conducted over six phases consisting of two study phases, a retrieval practice phase, a distractor phase, a final test phase and a recruitment phase. In the first study phase, participants were initially informed that they would need to make a recruitment selection decision between two applicants for a waitering job in a restaurant. The Curriculum Vitae (CV) of two candidates were displayed simultaneously on screen to confirm equal working experience and educational qualifications. Participants were assumed to detect two key differences in these CV’s that distinguished their ethnicity by nationality; an explicit statement of nationality and either a typical Japanese or Chinese name. Both names consisted of three Chinese characters. The CV’s were presented for 1 min and the on-screen position was counterbalanced for left–right presentation across participants. Next, participants memorized six positive and six negative traits of each applicant. Each trait was presented as a name-trait pair in the middle of the screen for 5 s with a 1 s interval time between name-trait pairs. To exclude the confounding of local interference effects, traits belonging to one candidate were never shown successively.

After learning all personal traits, participants were randomly assigned to one of two groups for the retrieval practice phase. In this phase, one group was required to recall only the negative traits of the Chinese applicant and the other group recalled only the negative traits of the Japanese applicant. Participants were provided with the applicant’s name and a phonological cue (using partial Chinese Pinyin) for each associated positive trait (e.g., LD is provided representing LANDUO which is the pronunciation of the Chinese word for lazy). Participants input their answer beneath the prompts. After retrieval practice, participants were administered a distractor task where they needed to select a preferred picture from a picture pair (an abstract picture vs. an impressionist picture). During this distractor phase, 30 picture-pairs were shown in consecutive order for 6 s each.

In the following recall (test) phase, participants were required to list all the memorized traits (positive and negative) according to their initial presentation by applicant name. Applicant names were presented on the top of the screen and the participants input the traits below the name, with the order of (name) presentation counterbalanced across participants. In the final (decision) phase, participants were required to state which applicant they would choose to employ and also to rate their confidence in this recruitment decision (from “1 = very unconfident” to “10 = very confident”).

#### Data analysis

The proportion of successfully recalled positive and negative traits were collated separately: RP+ refers to negative traits recalled (in the test phase) for the practiced applicant, RP− refers to the unpracticed positive traits (recalled in the test phase) belonging to the practiced applicant and BL refers to the positive traits belonging to the unpracticed applicant (recalled in the test phase). Recruitment decisions and confidence ratings were collated for each applicant. All data were analysed using frequentist and Bayesian statistics using JASP (JASP team, 2020; the outcome of the Bayesian analysis is the same as the frequentist and can be found here: https://osf.io/e9s8t/?view_only=b52482eba300473396293006adb195a5).

### Results and discussion

**Fig. 1:**
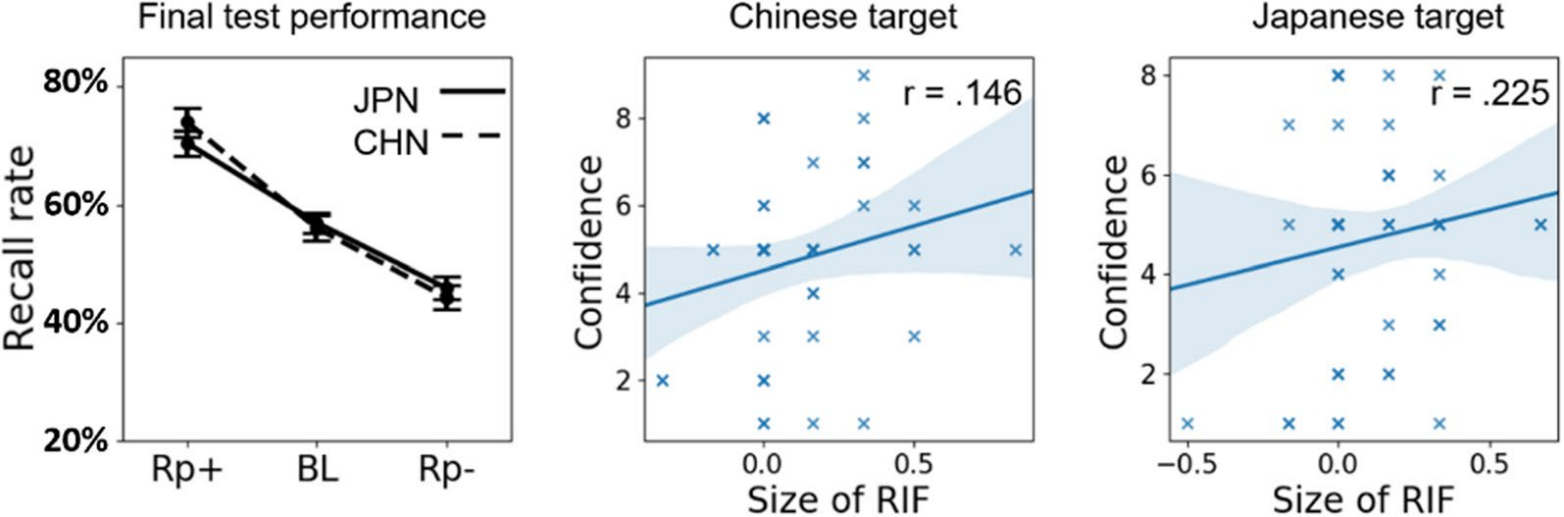
(Left panel) displays the final recall rate for RP+, RP− and BL traits for the Chinese (CHN) and Japanese (JPN) retrieval targets. Error bar indicates ± 1 standard error. (Middle panel and right panels) display the correlations between the size of RIF effect and the final selection confidence ratings for Chinese and Japanese target applicants. Markers in bold indicate more overlap in the data points

To determine whether retrieval-induced forgetting varied by applicant’s ethnicity, a 2 (ethnicity target: Chinese or Japanese) * 3 (trait type: RP+, RP− and BL) Analysis of Variance (ANOVA) was conducted on the final test recall rate (see Fig. 1, left panel). This revealed a significant difference between RP+, RP− and BL trait scores, *F*(2, 202) = 83.499, *p* < 0.001, *η*_*p*_^*2*^ = 0.452, but neither the main effect of retrieval target (*F*(2, 202) = 0.041, *p* = 0.83, η_p_^2^ = 0.000) nor the two-factor interaction (*F*(2, 202) = 0.881, *p* = 0.420, *η*_*p*_^*2*^ = 0.007) was significant. Planned comparisons revealed significantly more RP+ traits were recalled in the final test compared with RP− (*t* = 12.87, *p* < 0.001, *d* = 1.27) and BL traits (*t* = 7.48, *p* < 0.001, *d* = 0.737). More critically, a RIF effect was observed, as fewer RP− traits were recalled compared to the BL traits of the unpracticed person (*t* = 5.38, *p* < 0.001, *d* = 0.530).

For the final recruitment decision, most participants selected the Chinese applicant. There was no evidence that practicing negative traits of either the Chinese or the Japanese applicant altered the proportion of decisions to recruit the Chinese applicant (Chinese as target: 81.1%; Japanese as target: 82.0%, *χ*^*2*^ = 0.01, *p* = 0.91). The groups did not vary in the confidence of their selection of the Chinese applicant (Chinese target: *M* = 4.70, *SD* = 2.11; Japanese target: *M* = 4.74, *SD* = 1.96; *t* = 0.104, *p* < 0.91, *d* = 0.020). To determine whether there was an association between forgetting of traits (i.e., the effect size of RIF: the difference between BL and RP−) and the rated confidence of the recruitment decision, correlations were analyzed separately for the two retrieval targets (see Fig. 1, middle panel and right panels). No significant correlations were found for either retrieval target (Chinese target: *r* = 0.146, *p* = 0.30; Japanese target: *r* = 0.225, *p* = 0.11).

From these data, we found evidence of a generalized social RIF that disadvantaged the retrieval of (unpracticed) positive traits for a target applicant at test, compared to those baseline traits retrieved for a non-target applicant. There was no evidence that the RIF effect was moderated by the ethnicity of the practiced applicant. This result was consistent with Pica et al. (2016) who also reported no moderation of the social RIF when the task setting excluded any overt self-threat. Overall, Chinese participants favored the Chinese applicant in the recruitment decision and were reasonably confident in their choice. The opportunity to practice retrieving the traits of a target applicant (whether Chinese or Japanese) did not alter the participants’ final choice or their confidence. There was no association between the effect size of this social RIF and the participants’ confidence in their recruitment choice. In summary, there was no evidence that social RIF effects were moderated by a self-enhancing motivation, or by negative attitudes towards a Japanese applicant, even though negative attitudes towards a Japanese target applicant are widely-held in Chinese society.

To further understand the setting conditions for observing any influence of a motivated bias for ethnic categories within a social RIF paradigm, Experiment 2 used an identical experimental procedure to Experiment 1, but with the inclusion of a mild and distal threat to self-esteem.

## Experiment 2

### Method

#### Participants

One hundred and five Chinese nationality volunteers participated in the experiment. Participants received a souvenir medal as a gift for participation. All the participants gave consent to participate in the experiment and we did not adopt any exclusion criterion.

#### Materials and procedure

The materials and procedure were identical to Experiment 1 except that we added a manipulation of self-esteem threat to the self-threat induction task at the beginning of the study. Participants in the self-threat (Experiment 2) and non-self-threat conditions of this task (Experiment 1) received different feedback. No matter how many questions the participants had answered correctly, participants in the self-threat condition (Experiment 2) received the feedback “You have correctly answered 3 out of 12 questions. The average score is around 6”. This task was designed to induce uncertainty due to the ambiguous relation between their actual and stated performance, however, there was an additional concern for judging themselves in relation to other’s perceived performance that was absent in Experiment 1 and was designed to induce a self-protecting motivation, prior to the memory task.

### Results and discussion

A 2 (candidate being retrieved: Chinese or Japanese) * 3 (trait type: Rp+, BL and Rp−) ANOVA revealed significant difference between Rp+, BL and Rp− items, *F*(2, 210) = 220.24, *p* < .001, *η*_*p*_^*2*^ = 0.677. More Rp+ traits were recalled compared with traits in the BL (*t* = 9.89, *p* < 0.001, *d* = 0.956) and the Rp− condition (*t* = 20.98, *p* < 0.001, *d* = 2.03); more BL traits were recalled than Rp− traits (*t* = 11.09, *p* < 0.001, *d* = 1.07). The main effect of retrieval target was significant, *F*(1, 105) = 6.542, *p* = 0.012, *η*_*p*_^*2*^ = 0.059, but critically, the interaction between trait type (Rp+, BL or Rp−) was also significant, *F*(2, 210) = 10.54, *p* < .001, *η*_*p*_^*2*^ = 0.091. A simple effects analysis demonstrated that retrieving the negative traits for a Japanese candidate generated an additional disadvantage for the memory of their unpracticed Rp− traits, *t* = 5.16, *p* < . 001, *d* = 1.14.

**Fig. 2:**
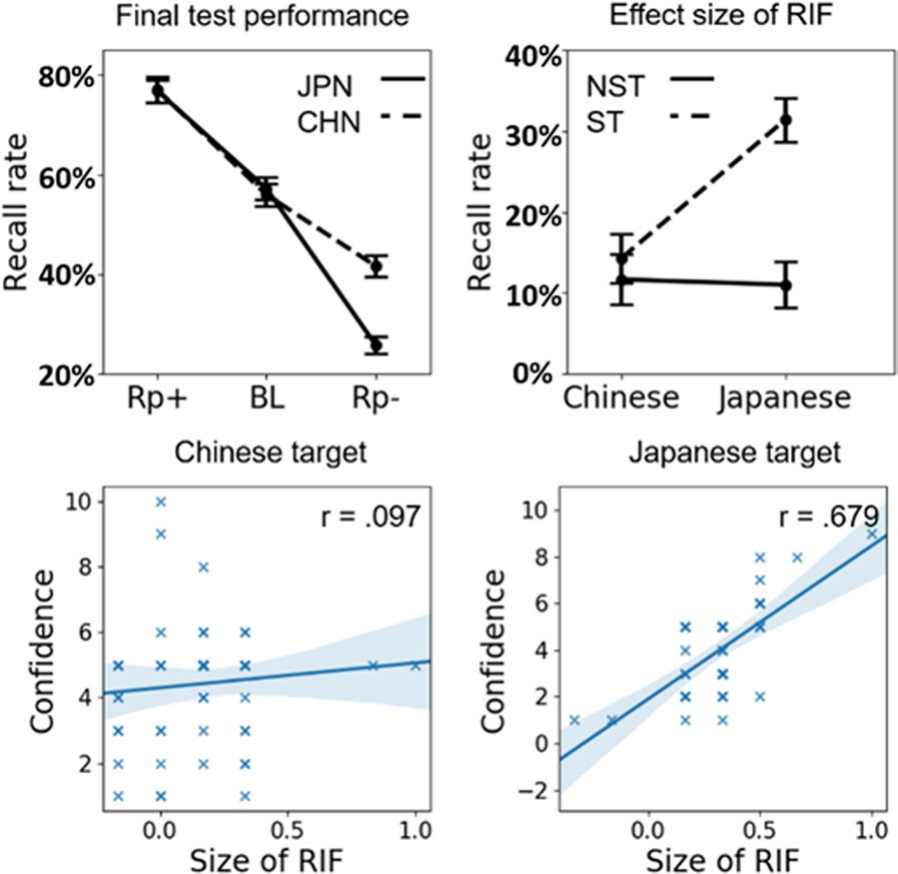
(Top-left panel) Recall rate of Rp+, Rp− and BL traits under Chinese (CHN) target and Japanese (JPN) target. Error bars indicates ± 1 standard error. (Top-right panel) Effect size of RIF under self-threat condition/non-self-threat and Chinese target/Japanese target condition. Error bars indicates ± 1 standard error. (Bottom left and right panel) Correlation between the size of RIF and final selection confidence. A darker shade indicates more overlapping data points

To address the role of self-threat more directly, we combined the data across Experiment 1 and 2, and conducted a further analysis using the size of RIF (BL—Rp−) as the dependent variable, and the target applicant and the self-threat condition as independent factors. The main effects of the target applicant (*F*(1, 206) = 7.836, *p* < 0.006, *η*_*p*_^*2*^ = 0.037) and self-threat (*F*(1, 206) = 15.34, p < 0.001, *η*_*p*_^*2*^ = 0.069) were significant. As is shown by Fig. 2 (top-right panel), the interaction was significant (*F*(1, 206) = 9.15, *p* < 0.003, *η*_*p*_^*2*^ = 0.043). An enhanced RIF was only found when retrieving the negative traits of Japanese candidate under a self-threat condition, (Experiment 2; *t* = 4.11, *p* < 0.001, *d* = 0.80).

Analysis of the participants’ final selection for the job revealed a similar pattern for each experiment. As in Experiment 1, the majority of participants selected the Chinese applicant. The decision to select the Chinese applicant did not vary with the ethnicity of the target applicant (Chinese as target: 78.4%; Japanese as target: 85.7%, *χ*^*2*^(1) = 0.01, *p* = 0.325) and there were no differences in participants’ confidence in their decision (*t*(104) = 1.26, *p* = 0.21, *d* = 0.244). However, unlike Experiment 1 (see Fig. 2, bottom-right panel and bottom-left panel), a significant correlation was found between the size of RIF and the confidence of the final selection, after practicing the negative traits for the Japanese target applicant (*r* = 0.679, *p* < 0.001). There was no association between the size of the RIF and confidence in selecting the Chinese target applicant (*r* = 0.097, *p* = 0.475). Confidence levels were moderately high and they did not approach ceiling for either applicant.

Finally, to consider the relationship between decision confidence and the memory scores, as suggested by correlations shown in Figs. 1 and 2, we further conducted a 2 (candidate being retrieved: Chinese or Japanese) * 2(self-threat: Experiment 1 or 2) Analysis of Covariance (ANCOVA) with the size of RIF as the dependent variable and confidence as a covariate. The result indicates a significant covariance of confidence with the RIF scores (*F*(1,205) = 15.90, *p* < 0.001, *η*_*p*_^*2*^ = 0.061). In addition, there was an adjusted main effect of candidate being retrieved (*F*(1,205) = 9.90, *p* < 0.002, *η*_*p*_^*2*^ = 0.038) and self-threat (*F*(1,205) = 20.804, *p* < 0.001, *η*_*p*_^*2*^ = 0.079). The two-way interaction (*F*(1,205) = 16.88, *p* = 0.001, *η*_*p*_^*2*^ = 0.052) remained significant, indicating an enhanced RIF for the out-group applicant under conditions of self-threat (*adjusted mean* = 1.88, *SD* = 1.77), compared to non-self-threat (*adjusted mean* = 0.66, *SD* = 1.22). This compared to only a slight enhancement of RIF for the in-group applicant under conditions of self-threat (*adjusted mean* = 0.857, *SD* = 1.38), compared to non-self-threat (*adjusted mean* = 0.70, *SD* = 1.298). Although this approach computes residuals by pooling the within-group regression the RIF scores on the confidence ratings, this approach confirmed that the findings of the interaction of RIF scores with threat conditions were relatively stable.

In sum, when manipulating self-threat in Experiment 2, the data revealed an interaction between the effect size of RIF and the ethnicity of the target applicant. An enhanced RIF was found when Chinese participants practiced the negative traits of the Japanese candidate in the context of self-threat. The finding confirmed that RIF effects can be moderated by motivational and contextual factors. This modified RIF emerged from a relative decrease in the recall rate for the unpracticed positive traits of the Japanese applicant, after practicing other negative traits of that person. In addition, the size of RIF significantly predicted confidence ratings of the final selection of the Chinese (in-group) applicant, but only in Experiment 2 (Japanese-target * self-threat condition). One explanation is that this conditional correlation relates to the cognitive processes elicited by the combination of self-threat and the need to recall negative traits of an out-group target. A more cautious interpretation is that the smaller effect size of the RIF in other conditions constrained the likelihood of observing a correlation with confidence ratings.

## General discussion

The present study hypothesized that retrieval-induced forgetting constitutes a cognitive pathway to inadvertent bias in decision-making. The study extended and refined previous work showing that RIF is malleable to cultural stereotypes by considering a well-established ethnic division between Chinese and Japanese people. Two experiments demonstrated a classic social RIF effect that providing practice at memorizing social traits with negative valence influences the forgetting of positive traits that were unpracticed (for the non-target applicant). Yet, ethnic divisions between in-group and out-group targets influenced the size of the RIF effect when participants were placed under conditions of mild threat to their self-esteem in Experiment 2. This was consistent with previous findings of a modified ethnicity-RIF linked to cultural stereotypes of African Americans and Asian Americans in Western context (e.g., Dunn & Spellman, 2003; Experiment 3; Pica et al., 2017), but critically, the pattern was only observed with Chinese citizens under conditions of self-threat in the current study.

A key question is how to interpret the presence and absence of ethnicity-RIF effects where self-threat was an enabling factor for observing the enhanced ethnicity-RIF effect. Like Pica et al., (2018a, 2018b), we conclude that RIF effects are modified primarily by motivational factors linked to self-protection. In the current design, RIF scores in the critical condition of practicing the out-group target, include orienting to the negative traits of this target, and retaining the positive traits of the Chinese in-group target, to maximise memory performance. It is likely that inhibitory process linked to self- protecting motivation guided retrieval. As an enhanced RIF score, the positive traits of the in-group target were suppressed. However, we did not find a diminished RIF that protected the in-group positive traits from forgetting, that is also consistent with a self-protecting motivation that serves to restore self-esteem of the in-group. This enhanced RIF effect was conferred by *suppressing*, rather than inflating, the positive traits of the Chinese (in-group) target as the baseline. As the negative traits of the out-group target are practiced and readily retrieved at test, one possibility is that inhibitory processes are directed to resolve competition with the positive traits, to retain the negative attributes of the out-group. The inhibition account of RIF holds that inhibition processes operate in a nuanced way towards maintaining performance at test to retain the practiced items. If cultural concepts associated with the out-group target are activated as an additional source of competition at test; this could induce a cost of resource allocation that falls on the memorization of the unpracticed (positive) traits of the in-group target. This is related to Dunn and Spellman’s finding that a stronger commitment to stereotypic belief (that engages cultural concepts relating to the out-group target) reduced the size of their RIF effect, despite preserved inhibition of unpracticed stereotypical traits.

A motivational account based around a self-protecting motivation focusses on the relative costs to the individual and their performance on the memory task in situ. In doing so, we place less emphasis on an explanation that aligns solely with stereotype activation by emphasizing the maintenance and retention of negative attributes of the out-group target in all settings. Within our sample population of Chinese citizens, there was a high likelihood that stereotype activation would be conferred on a Japanese applicant for a job. However, a stereotype activation account would confer different patterns of performance. If stereotyping draws from the retention of more negatively-valenced concepts overall, then both experiments could be expected to show an enhanced in-group RIF at test, corresponding with more accessible negative traits of Japanese people stored in memory. However, there was no indication of this, only an enhanced out-group RIF (by suppressing positive in-group traits) for Experiment 2. Therefore, self-protecting motivations played a role, possibly in how participants attended to the memory task, or allocated resources to favour the retrieval of negative attributes of the out-group. Although self-enhancing and self-protecting motivations are pan-cultural, their behavioral expression can be culturally diverse (Heine, 2005; Heine & Hamamura, 2007; Heine et al., 1999; Kitayama et al., 1997). In China, self-enhancement motivation can directly influence memory for traits associated with one’s ethnic in-group, with several studies showing faster and stronger memorization of in-group traits for Han Chinese compared to Chinese minority groups (Mamat et al., 2014; Yang et al., 2008). It is notable that our Chinese participants were able to suppress positive in-group traits to generate the enhanced RIF. Whilst motivated cognition can influence memory for traits of Chinese people, there is a risk that cultural-specificity in the expression of these motivations could influence when prejudice occurs in memory and decision-making practices.

Although the present study together with Pica and colleagues’ work converges on the significance of motivational factors, our data does not entirely mirror previous findings of motivational influences on RIF. First, our ethnicity-RIF was robust when practicing the negative traits of the out-group (Japanese) target under conditions of no self-threat, similar to the equivalent RIF for practicing negative traits of the out-group (African-American) target under corresponding conditions in Pica et al. (2017). In their study, the RIF was eliminated for the out-group under conditions of practicing the positive traits of in-group members, so that negative traits of the out-group were protected from being forgotten, even under conditions of no self-threat. Although the RIF is clearly modified by motivational factors, the basis for either enhancing or elimination of the RIF needs further work to test the conditions under which the ethnicity-RIF if modified. Second, we demonstrated a significant enhanced RIF of the positive traits belonging to the Japanese candidate, while the corresponding effect in Pica et al. (2016) was not quite significant (*p* = 0.09). This inconsistency might be due to a smaller sample size of Pica and colleagues’ work, leading to less power. For each condition, only 15 participants were recruited so the statistical power of their analysis was only 0.63, whereas our studies achieved a power of 0.98. There could be different flavors of a modified ethnicity-RIF including those that correspond to motivational factors and elicit self-enhancing activity, or those that correspond more directly to stereotype activation and change the accessibility and content of concepts retrieved.

The current study sought more generalisability to the interpretation of these RIF effects by extending to a homogenous sample of Chinese citizens who comprise a dominant majority, but whose cultural stereotypes are formed outside those studied within Western society. In this sense, the participants decisions regarding the employability of the target applicants were possibly more selective to Chinese preferences. In these young Chinese people, a strong in-group bias was detected, favouring the selection of the Chinese target as an employee. On one hand, this in-group preference could be independent of the memory task. If there is a strong cultural expression of social conformity and collective endeavour within Chinese society, then one consequence could be that in-group applicants are openly favored and actively selected. However, employment discrimination is a global phenomenon. China has clear statements of law against ethnic discrimination within the employment sector (People’s Republic of China, Article 12; Labor Law and Article 13; Promotion of Employment Law) and some evidence points to lower levels of employment discrimination against Chinese minority individuals in state-owned firms (Maurer-Fazio, 2012). Collectivist cultures cannot be simply aligned with in-group favouritism, rather, a cultural expectation of reciprocity could contribute to rewarding in-group members (Yamagishi et al., 1998). In the present study, we found a stronger correlation between the size of RIF and the confidence of Chinese people in their decision. This was only detected when recalling negative traits of the Japanese candidate under a self-threat condition, implying that self-protecting motivations could potentially determine the probability of people’s decision bias in conjunction with the accessibility of personal traits in memory. Collectivism does not confer strong motivations for bias, rather, personal motivation does appear to be a significant factor in the present study.

### Limitations of the study

The current study has several limitations. One limitation of the present study is that we did not test the ethnicity-RIF effect with a different target valence (i.e., retrieving positive traits of the applicants). This leads to an unbalanced design regarding the role of valence, and limits comparison of ethnicity-RIF effects with different out-groups in the presence and absence of threat (e.g. Pica et al., 2017). However, the framing of negative information about ethnic minority targets is a particularly important and sensitive aspect of discussion in the context of employment activity, such as interviews or promotions procedures. A second limitation of our ethnicity-RIF was the design of the final recall test, as a category-cued design, rather than a category-plus-specific cue design. Interference effects are more likely (see Schilling et al., 2014), suggesting that forgetting was not a pure reflection of inhibitory control. Future work should address the ethnicity-RIF through the consequences of inhibitory processes that are invoked when discussing positive traits of in-group applicants, using a purer measure of these processes at final test. Similarly, the between-group manipulation of target ethnicity could be considered more carefully regarding the participant characteristics, including explicit attitudes towards the out-group. Other limitations of the study relate to the use of the self-threat induction task to elicit motivated cognition. Since we did not explicitly check this manipulation, it is not clear whether participants actively believed the feedback that was given in both experiments. It is likely that participants responded to ambiguity of feedback given the absence of exact test scores and that this contributed in some way to their self-efficacy on the memory task, however their exact beliefs and timing of the induction task should be explored further.

### Disrupting systemic racism

Our findings clarify how cognitive processes relating to memory and decision-making can be instrumental to work-place decisions. We highlight that RIF is not only a cognitive mechanism, but it can be influenced by people’s attitudes and motivation towards ethnic minority groups. Specifically, we show (a) that ethnicity of a minority group modifies the RIF when people from a majority ethnic group have received ambiguous feedback and are able to reference their own poor performance relative to others, and (b) that this modified-RIF relates to confidence of the majority group when making an employment decision. Together, this implies that meta-cognitive awareness of one-self as comparing less favourably to other people, is critical to how modifications are made to RIF effects, and how these modifications (to the RIF) then apply to their confidence when making decisions. Our design emphasized the practice of negative traits of in-group and out-group candidates for a job. This is a sensitive and challenging aspect of recruitment, where majority in-group members may feel uncomfortable with explicit discussion of minority applicants in lieu of their legal and moral responsibilities to fair recruitment. Yet, comparing positive and negative traits of candidates for recruitment and promotion can be a necessary aspect of the decision-making process. For example, personal characteristics and traits can be actively discussed before, during and after interview to compare different candidates. Our ethnicity-RIF findings suggest that rehearsing (practicing) these personal traits can place minority candidates at risk in several ways. First, any modification of the RIF will impact how information is retained and forwarded to informational schemas about the minority candidate. That is, we found negative traits were retained and the positive traits of out-group candidates were suppressed in the enhanced ethnicity-RIF in Experiment 2. If the RIF operates more definitively to protect retrieval of the practiced negative traits of minority candidates, then this is likely to continue to shape a shared narrative around negative traits of that person, when this information is socially exchanged. Finding ways to limit the implications of an enhanced (or reduced) RIF for social exchange about minority candidates is necessary, and the costs and benefits of discussing their positive traits should be acknowledged too. Disrupting systemic racism is to be wary that the conversation around negative traits of a minority candidate does not persist longer than is necessary, or that discourse does not recourse back to more rehearsal of negative traits, due to poor regulation of forgetting.

Second, our findings point to the significance of personal esteem, or how people protect their own sense of self-worth. We found modified forgetting only when people were subject to conditions that would arouse a self-protecting motivation (in this case, direct evidence that their performance was below par). Recruitment or promotion opportunities are highly likely to induce self-other comparisons, particularly regarding how people perceive their performance in their role, or within the workplace generally. This tension can only be highlighted by the need of recruiters, or promoters, to recognize a new ‘other’ in the workplace, who may bring different experiences and qualities, or favoured in different ways. Even the exposure to employment decision-making could increase the need for self-protection for the recruiting individual, despite their apparent security, seniority or experience in the role. Disrupting systemic racism is to actively offset the risks of self-protecting motivations that will modify forgetting to sustain negative traits of minority applicants. Simple tools such as reminding people of their competence in their current role could be undertaken immediately before discussion of the negative traits of minority applicants, could offset this risk.

Third, our findings point to an association between these meta-cognitive aspects of forgetting and people’s confidence in decisions made. We acknowledge that the selection bias was stable in our data. To combat systemic racism, the metacognitive aspects of decision-making need to be recognized. Dismantling racism by ranking the certainty of decisions, alongside the decision preference, could generate opportunities for ethnic minority candidate to be more actively considered in the final decision, rather than using preference data alone to rank different candidates.

Overall, the present study addressed the contemporary understanding that RIF is not only a cognitive effect, but it can be influenced by people’s attitudes and motivation. Importantly, this effect appears to be sensitive to mild levels of perceived self-threat. Arousing self-protecting (or other) motivations are particularly salient to memory modification for ethnic minority persons within the fragile environment of workplace decision-making. We generate three specific suggestions to disrupt systemic racism in the context of employment decisions about ethnic minority candidates. First, to limit and review, rather than avoid, a discourse around negative traits of minority applicants in interview settings. Second, to remind recruiters of their competence in the workplace, to offset the risk of self-protecting motivation. Third, to consider practical ways in which metacognitve aspects of decision-making, rather than preference, can be used to rank candidates. This could be a fruitful way to protect people who consistently suffer from the collective failure of the dominant majority ethnic group to identify and resist inadvertent bias.

## Data Availability

The datasets used and analysed during the current study are available in the OSF repository, https://osf.io/j8cy4/?view_only=b41f0e8ae02748b8a32f710e7f3e5ce1. Summary data from the pilot study and Bayesian analysis are available in the OSF repository; https://osf.io/bemnh/?view_only=b52482eba300473396293006adb195a5.

## Abbreviations

RIF: Retrieval-induced forgetting
