# Correction: Self-protecting motivation, indexed by self-threat, modifies retrieval-induced-forgetting and confidence in employment decision bias against out-group targets

**DOI:** 10.1186/s41235-022-00393-7

**Published:** 2022-05-13

**Authors:** Shaohang Liu, Christopher Kent, Josie Briscoe

**Affiliations:** School of Psychological Science, University of Bristol, 12a Priory Road, Clifton, Bristol, BS8 1TU UK

## Correction: Cognitive Research: Principles and Implications (2021) 6:77 https://doi.org/10.1186/s41235-021-00334-w

The original article Liu et al. (2021) contained a typo in co-author, Shaohang Liu’s name which has since been corrected.
